# Tracing the Growth, Gaps, and Characteristics in Positive Education Science: A Long-Term, Large-Scale Review of the Field

**DOI:** 10.3389/fpsyg.2021.774967

**Published:** 2021-12-03

**Authors:** Lea Waters, Daniel Loton

**Affiliations:** ^1^Centre for Wellbeing Science, Melbourne Graduate School of Education, University of Melbourne, Melbourne, VIC, Australia; ^2^Connected Learning, Victoria University, Melbourne, VIC, Australia

**Keywords:** student, education, mental health, well-being, positive psychology, positive education

## Abstract

This large-scale quantitative review used publication data to track the presence of positive education terms over a 100+ year period across 35 psychology journals and education journals utilizing two analytical methods. First, computer-generated linguistic word count analysis identified that positive education terms have shown small but steady growth in psychology and education research for more than a century. From 1904 to 2016, positive education terms have risen consistently, with increases in 1952, 1982, 2010, and 2014 to over 4, 5, 6, and 7 percent, respectively. Four new terms were present in the top 20 most prevalent terms following the official launch of positive education in 2009: well-being, satisfaction, motivat*, and engag* (note: terms ending with an asterisk are word stems). Three terms also increased in rank order prevalence from 2009 onwards: emotion*; health; and goal*. The second analytical method involved in-depth human coding of a subset of positive education abstracts (*n*=2,805) by a team of five researchers^1^ to identify trends pertaining to how positive education research has been conducted in terms of paradigms, designs, methods, tools, samples, and settings from 1950 to 2016. College students and students in secondary school make up the most common samples, with little research in the early childhood years. Quantitative, cross-sectional studies using self-report surveys have been the most common design and method used over the past six decades, suggesting room for growth in qualitative methods and the need for greater longitudinal and intervention designs. The human coding was also used to classify positive education variables into broader categories of research. Nine categories were identified: positive functioning; well-being; ill-being; strengths; agency; connection and belonging; identity and personality; school climate and outcomes; and demographics. By tracking positive education science over time, the current paper allows researchers to take stock of the field, identify gaps, outline areas of growth, and pursue fruitful topics for future research.

## Introduction

Student mental health has become a global priority in education (WHO, 2013; UNESCO, 2015; WHO and UNESCO, 2021). The Organisation for Economic Co-operation and Development (OECD, 2015, p. 32) suggests that “[p]erhaps the ultimate goal of education policy makers, teachers, and parents is to help children achieve the highest level of well-being possible.” This aim is reflected in inter-governmental initiatives, such as “Focusing Resources for Effective School Health” (FRESH), developed through cooperation between WHO, UNESCO, the World Bank, and United Nations Children’s Fund (UNICEF) in 2000 (WHO et al., 2000; Wikipedia, 2021) and the “Health Promoting Schools” guidelines released as a joint collaboration by WHO and UNESCO (2018; United Nations, 2021).

While inter-governmental initiatives to promote student well-being have become more widespread over the past decade, these aims are not entirely new. Indeed, mental health interventions for students have been researched for more than a century (Witmer, 1897; Hildreth, 1930). The current review paper has four aims: (1) to investigate whether education-based mental health research has grown over the past century from 1904 to 2016; (2) to identify the top positive education terms that have received research attention from 1950 to 2016 and to examine trends in the proportion of these terms over time (i.e., *what* has been studied in positive education); (3) to identify the research paradigms, designs, tools, samples, and research sites most commonly used in education-based mental health research from the 1950s to 2016 (i.e., *how* has positive education been studied); and (4) to examine whether the official launch of positive education (Seligman et al., 2009) has led to a higher number of well-being-oriented, positively focused topics being studied compared to research prior to this time.

### Student Mental Health Interventions: The Evolving Evidence Base

Broadly speaking, early educational mental health interventions were deficit-oriented and concerned with “treating the many difficult cases that resist the ordinary methods of the school room” (Witmer, 1897, p. 117). Over time, however, intervention programs have expanded beyond a focus on *targeted treatment* to also include *universal prevention* and, more recently, *mental health promotion* (Peterson and Park, 2003; Froh et al., 2008). Prevention programs initially targeted students at risk of developing mental disorders – that is, students identified as struggling and/or with subclinical symptoms (Herman et al., 2004). However, these were later expanded to include all students through universal approaches following the logic that prevention-oriented skills can serve as a potential “antidote to depression” for everyone (Seligman et al., 2009, p. 295). Universal mental health prevention programs are run with large groups of students regardless of risk factors or where they sit along the mental health continuum (Fenwick-Smith et al., 2018). These programs teach general skills designed to help students buffer against distress and stave off psychological illness (Jaycox et al., 1994; Pattison and Lynd-Stevenson, 2001).

Seligman et al. (2009, p. 295) argued that universal mental health interventions are not only means to prevent student distress but can also function as a “vehicle for increasing life satisfaction.” Their call formed part of a further evolution in the way educational institutions approached student mental health – this time through the adoption of *promotion programs*. Promotion programs emphasize creating curricula that bolster the positive end of the mental health curriculum as opposed to reducing the negative (Waters, 2011; Waters et al., 2017). A recent review of 221 universal positive psychology interventions (PPIs) in schools by Owens and Waters (2020) from 2010 to 2018 found prevention programs were slightly more prevalent than promotion programs (18 percent compared to 16 percent); however, they also found that the majority of programs had a dual aim of prevention *and* promotion (67 percent) by teaching skills that reduce ill-being, such as cognitive reframing, combined with skills that promote well-being, such as savoring and strengths use, and by seeking to reduce indices of ill-being (e.g., anxiety) and increasing aspects of well-being (e.g., life satisfaction).

In addition to looking at the target of programs being implemented (i.e., treatment, prevention, promotion), a brief review of the history of education-based mental health interventions reveals they have shifted through various psychological schools of thought (Dawood, 2013). For example, in the 1980s and 1990s, three notable approaches came to the fore: student coping programs (Stevens and Pihl, 1983; Schinke et al., 1987), social-emotional learning (SEL; Ladd and Mize, 1983; Creemers and Tillema, 1987), and resilience education (Stevens and Pihl, 1983; Schinke et al., 1987; Cowen et al., 1990). These approaches were primarily situated in the prevention space and aimed at helping students to reduce stress, anxiety, and depression (Reivich and Gillham, 2010).

Beginning in the early 2000s, there has been a notable growth in promotion programs aimed to increase the positive end of the mental health continuum (Keyes, 2002) and adopting positively oriented approaches, such as values education (Nielsen, 2005), character education (Berkowitz and Bier, 2005), civics education (Cogan and Morris, 2001), positive youth development (Larson, 2000), mindful education (Wall, 2005), and positive education (Seligman et al., 2009). While each approach has a slightly different orientation and focus (for an explanation of the emphasis and differences between each, see Waters et al., 2017), all can be grouped under the broad umbrella of positive psychology given that they aim to move beyond symptom amelioration to also focus on building universally applicable positive capacities in students.

### Positive Psychology and Its Role in Education

Launched in the late 1990s as a way to counterbalance deficit-oriented research in psychology (Seligman, 1999), positive psychology (PP) calls for scientific inquiry into positive traits, states, processes, and capacities that help to strengthen mental health, maximize potential and cultivate pro-socialness (Seligman and Csikszentmihalyi, 2000; Sheldon and King, 2001). In line with the view that a full state of mental health involves more than the absence of illness (Keyes and Lopez, 2002), PP focuses on the promotion of well-being – beyond the treatment of ill-being – and aims to assist people, groups, and institutions to flourish (Aspinwall and Staudinger, 2003; Gable and Haidt, 2005). Starting initially within the field of psychology, the call for positively oriented research soon spread to other fields such as business, sport, and education (Rusk and Waters, 2013; Donaldson et al., 2015).

In terms of education, PP can be seen to have played a significant role in shaping education-based mental health research since the 2000s in three key ways (Clonan et al., 2003; Salmela and Uusiautti, 2015). First, research from PP has been used to reshape treatment programs to also include a strength-based approach and adopt the premise that resilience and strengths can be unlocked in struggling students given the right support (Steck et al., 2003; Prout, 2009). Second, early findings coming from PP helped schools to expand their prevention-oriented programs to include lessons that promoted positive attributes and outcomes (Lubinski and Benbow, 2000; Akin-Little et al., 2003). Third, given that positive psychology could be taught to all students, not just those who are at risk or are in need of remediation, it widened mental health promotion programs to become whole-school approaches (Miller et al., 2009; Allen et al., 2018b). In these three ways, we can see how the arrival of PP in 1999 has shaped education-based mental health research since the 2000s.

Adding to the above, PP further consolidated its role in education-based mental health research through findings showing that aspects of PP (e.g., hope, character strengths, and gratitude, to name a few) were predictive not only of student mental health but also of academic achievement (Snyder et al., 2002; Austin, 2005; Froh et al., 2008). Moreover, and notwithstanding moderators of intervention effects that require further study, PP interventions can be applied across multiple student contexts and practiced outside of the school (e.g., Huppert and Johnson, 2010), making them relatively accessible and scalable (Chafouleas and Bray, 2003). Finally, PP was shown to be applicable to teachers and faculty (McGovern, 2011; Critchley and Gibbs, 2012), which is important given that teacher well-being is associated with a range of student outcomes, including well-being and academic results (Oberle and Schonert-Reichl, 2016). Finally, positive psychology research has also adopted ecological theories of well-being and organizational change models to inform whole-school and whole-university change (Hoy and Tarter, 2011; Oades et al., 2011a,b), thus further bolstering its place in shaping education-based mental health research.

The application of PP to education was given the name “positive education” by Seligman et al. (2009, p. 294), who argued that educational institutions should “teach both the skills of well-being and the skills of achievement.” More recently, Waters and Loton (2019) described positive education as a field that weaves contemporary knowledge from the science of well-being into educational practice.

While initial studies identified as positive education distinguished themselves from earlier education interventions by focusing on the teaching of specific PP skills, such as gratitude or hope to promote the positive end of the mental health continuum (see Waters, 2011), the boundaries between positive education and other movements are often blurred (Kristjánsson, 2012). For example, Quinlan et al. (2014) conducted an intervention on character strengths that was published in the *Journal of Positive Psychology*. This intervention was situated within the positive education literature but is also clearly an intervention that falls within the field of character education. Similarly, Huppert and Johnson (2010) evaluated the impact of a mindfulness intervention on psychological well-being and published it in the *Journal of Positive Psychology*. This intervention is considered to be positive education because of its aim to promote well-being (many other mindfulness interventions focus on reducing anxiety and stress; see Waters et al., 2015 for a review of mindful intervention in education) but it is also clearly an intervention that falls within the mindful education movement. Moreover, while both of the interventions mentioned above were classified by the authors as positive education studies, they align closely to the positive youth development movement. Our intention with these examples is to show that the education-based mental health movements arising in the 2000s are more common than they are different and, for the purpose of the current review paper, rather than draw boundaries between these movements, we will adopt a broad net to include all education-based mental health research that is positively oriented as belonging to “positive education.”

### Existing Positive Education Reviews

Positive education has been heralded as a fast-growing field of science (Shankland and Rosset, 2016; Chodkiewicz and Boyle, 2017). This expansion has motivated researchers to take stock of the field as a whole: the breadth, direction, key topics, and methods used. Review papers on positive education can provide a cumulative synthesis of knowledge on the conditions and processes that promote student well-being. Additionally, from the perspective of prioritizing future science, these reviews can be used to identify research trends and gaps in terms of the constructs studied and also in terms of research designs employed.

Prior to describing the current review, it is worthwhile examining the existing positive education reviews to learn about what the reviews focused on, how they were conducted and what scope and timelines have been used to ensure that a new review is adding additional knowledge. To date, there have been 17 reviews conducted on various facets of positive education published since its inception in 2009. These review papers align with Owens and Waters’ (2020) criteria that positive education research includes well-being skills and/or well-being indicators. The details of these review papers are outlined in Table 1.

**Table 1:** Existing positive education review papers.

Author and year	Focus	Type of review/method used	Data source	Sample	Time span of review	Data set	Sample size (*n*=Articles)
Brunwasser et al. (2009)	Penn Resiliency Program	Meta-analysis	PsycINFO	Students	1990–2009 (10years)	Published RCT studies	17
Durlak et al. (2010)	After-school SEL programs	Meta-analysis	PsycINFO plus three targeted journals	Students	1980–2007 (27years)	Published intervention studies using RCT designs United States only	75
Durlak et al. (2011)	SEL programs	Meta-analysis	PsycINFO plus 11 targeted journals	Students	1970–2007 (30years)	Published peer-reviewed intervention studies	213
Waters (2011)	School-based interventions covering resilience, gratitude, serenity, character strengths and hope	Qualitative review	PsycINFO	Students	2007–2011 (4years)	Published peer-reviewed articles	12
Froh et al. (2011)	76 positive education constructs	Content analysis: text search using a list 76 positive psychology terms	Four targeted journals	Information not provided	1960–2008 (48years)	Published peer-reviewed articles, intervention studies, basic research, applied research	1,168
Kristjánsson (2012)	Search for positive psychology topics in education	Critical review: text search	One targeted journal	Information not provided	2002–2012 (10years)	Published peer-reviewed articles	Information not provided
Meiklejohn et al. (2012)	School-based mindfulness interventions	Systematic review	Information not provided	Teachers and students	2005–2010 (5years)	Published peer-reviewed articles	Three teachers, 14 students
Waters et al. (2015a)	Contemplative education interventions (i.e., mindfulness, yoga, meditation)	Systematic review	Web of Science database	Students	1989–2011 (22years)	Published peer-reviewed articles, intervention studies, and case studies	15
Taylor et al. (2017)	Positive youth development and SEL programs	Meta-analysis	PsychINFO, Dissertation Abstracts, and Medline + ten targeted journals	Students	1970–2014 (4years)	Published peer-reviewed intervention studies and published reports	82
Maynard et al. (2017)	Mindfulness	Systematic review	Australian Education Index, British Education Index, CBCA Education, Education Complete, ERIC, MEDLINE, ProQuest Dissertations and Theses, PsycINFO, Social Science Citation Index, Social Service Abstracts, Sociological Abstracts, SPORTDiscus	Students	1990–2016 (26years)	Published peer-reviewed articles, unpublished studies, conference abstracts and proceedings, and other gray literature	61
Shankland and Rosset (2016)	Brief school-based PPIs that focused on mindfulness: gratitude, strengths, and positive relationships	Conceptual review	Information not provided	Students	2005–2015 (10years)	Published peer-reviewed papers, review papers, interventions studies, and cross-sectional studies	16
Allen et al. (2018a)	School belonging	Meta-analysis	PsycINFO and file drawer	Students	1993–2013 (20years)	Published peer-reviewed articles and file correlational and longitudinal studies	51
Waters and Loton (2019)	School-based interventions covering strengths, emotions, awareness, coping, habits and goals	Systematic review	Scopus, Google Scholar, PsycINFO, Web of Science. ancestry method	Students	1987–2016 (29years)	Published peer-reviewed intervention studies	75
Kumar and Mohideen (2019)	Character strengths	Scoping review	EBSCO, JSTOR, PubMed, Google Scholar, ProQuest, and ScienceDirect	Students	2000–2018 (18years)	Original published research or theses/dissertations	13
Owens and Waters (2020)	PPIs in schools	Qualitative review	PsycINFO, ancestry method; Google Scholar’s forward search option (“cited by”) was used to find newer articles that cited the older articles found	Students	2010–2018 (18years)	Peer-reviewed articles	212
MacCann et al. (2020)	Emotional intelligence	Meta-analysis	ERIC, Google Scholar, ISI Web of Science, Medline, ProQuest Dissertations and Theses, PsycINFO, PubMed, ScienceDirect, and Scopus	Students	2004–2016 (12years)	Published studies, unpublished data, test manuals, dissertation, and conference presentations	162
Lavy (2020)	Character strengths	Integrative overview	Google Scholar, Scopus databases; ancestry method using reference lists of strengths interventions and *VIA* Institute character strengths publications	Students	2011–2015 (4years)	Published intervention studies using RCT designs	Four

The existing reviews on positive education provide useful data about the topics that have gained focus in positive education. To date, these include resilience, SEL, coping, mindfulness, strengths, gratitude, hope, emotions, emotional intelligence, positive relationships, school belonging, and habits and goals. Fifty-eight percent of the reviews conducted in positive education focused on single topics such as emotional intelligence (e.g., MacCann et al., 2020) or character strengths (e.g., Lavy, 2020); 27 percent reviewed multiple topics that were preset (e.g., Shankland and Rosset, 2016 reviewed mindfulness, gratitude, strengths, and positive relationships); and two reviews did not start with a predetermined set of topics, but instead searched with a broad net to find all positive education topics (Froh et al., 2011; Kristjánsson, 2012).

Beyond identifying core topics in positive education (i.e., topics that have had a large enough number of studies to warrant a review), the existing review papers also provide information about the samples, timelines, and scope used to provide a big picture of positive education. With regard to samples, 88 percent of the reviews focused on students, with one review also including teachers, and two studies not specifying. A look at timelines shows that the review periods ranged from 4years to 48years, with the majority of reviews looking at a period of 10years or less (41 percent). With regard to the scope, the number of papers included in the review data sets ranged from four to 1,168 (mode=17; median=56; mean of 137.06±283.54), with 75 percent of the review studies containing less than 100 papers. In terms of method of analysis used in the review papers, the three most common types of reviews were meta-analysis (35 percent), systematic reviews (24 percent), and qualitative reviews (12 percent). Of relevance to the current paper, two studies used the analytical method of text analysis (i.e., Froh et al., 2008; Kristjánsson, 2012) to trace the growth of positive education over time and to identify the topics that have received focus.

The review papers conducted to date provide useful information about the growth and impact of positive education. This information is timely given the increased focus on student well-being in the past two decades and the impact of the COVID-19 (SARS-CoV-2) pandemic on student mental illness (Marques de Miranda et al., 2020). However, given that the bulk of the existing reviews focused only on a small range of preset specific topics or interventions, much of the field of positive education has not yet been represented in research syntheses. Add to that the reasonably small data sets (the majority contained less than 100 studies) and it is clear that previous review papers have been restricted from presenting a view of the full scope of positive education research. Finally, as existing reviews utilized relatively short timelines (the majority of reviews looking at time periods of 10years or less), there has yet to be an analysis of how the earlier school-based mental health research has shaped the current science, how positive education research has (or has not) grown over time, and whether the official launch of positive education in 2009 triggered a higher rate of well-being-oriented, positively focused topics being studied. Further reviews are needed to provide a longer-term and more comprehensive oversight of this growing field.

These gaps have motivated the current review paper, which seeks to provide a long-term big picture overview of positive education. To accomplish this, a review method is needed that can sift through and synthesize the large array of research topics and methods in positive education that have been studied over time. While the review methods that have most commonly been used in positive education to date are meta-analysis and systematic reviews, these methods use predetermined categories (i.e., meta-analysis starts with a preset lens on certain positive education interventions and/or specific outcomes to quantify their magnitude of impact *via* effect sizes) and are not able to handle very big data sets (e.g., findings from systematic analyses are typically researcher coded and, thus, utilize relatively small data sets). As such, we followed Froh et al.’s (2008) and Kristjánsson’s (2012) use of the analytical method of text analysis – but did so with a much larger sample. The current review method combines language analysis of a large corpus of psychology and education journal articles to identify *what* is being studied in positive education with a more detailed human analysis of a smaller but substantial subset of positive education abstracts to determine *how* positive education has been studied. The language analysis broadly aligns with bibliometric studies (see Donthu et al., 2021 for a guide), which have been used to examine very large fields of research, including PP (Rusk and Waters, 2013). Language analysis paired with machine learning is increasingly used to study varied aspects of well-being (D’Alfonso, 2020). The human analysis component aligns broadly with systematic reviews, especially those that focus on abstracts to identify study design characteristics (see, for example, Acosta et al., 2001).

## Materials and Methods

### Step 1: Sample

The current study followed the method used by Durlak et al. (2010, 2011), Froh et al. (2011), Kristjánsson (2012), and Taylor et al. (2017) of selecting targeted journals to create the database.^2^ The researchers above identified specific journals in a range of fields, including education, school psychology, child psychology, adolescent psychology, developmental psychology, prevention psychology, clinical psychology, and community psychology. The same method was used in the current study to identify the data set from within which the abstracts could be reviewed to investigate research trends in positive education. The first step was to review the journals used by the above authors and determine which of those were to be included in the current database. From the existing review papers, we included the *Journal of Consulting and Clinical Psychology*, *Child Development*, *Journal of Adolescent Research*, *Journal of School Psychology*, *Psychology in the Schools*, *School Psychology Review*, *School Psychology*, and *Educational Psychologist*. The next stage was to expand the list by going through the references of review papers outlined in Table 1 to determine whether there were any other journals that were frequently mentioned by these positive education reviews. Following this, relevant journals from the newer fields of PP and well-being science were also included.^3^ Finally, the draft journal list was sent to five positive education experts^4^ who were asked to review the list and recommend addition journals. In total, 35 journals formed the database for this study (see Table 2).

**Table 2:** Journals selected to form the database for current review paper.

Journal	Abstracts
**Educational journals**	
Journal of School Psychology	1,650
Psychology in the Schools	2,679
School Psychology Review	1,531
School Psychology	297
Educational Psychologist	756
Journal of Character Education	140
Educational Research Review	150
School Mental Health	184
Journal on Educational Psychology	233
Contemporary Educational Psychology	1,235
Educational Psychology	1,238
British Journal of Educational Psychology	2,180
Journal of Educational Psychology	8,854
**Positive psychology/well-being journals**	
Psychology of Well-Being	39
International Journal of Qualitative Studies on Health and Well-Being	286
The Journal of Positive Psychology	425
Journal of Happiness Studies	735
**Child, adolescent and development psychology journals**	
Applied Developmental Science	330
Journal of Applied Developmental Psychology	1,220
Developmental Science	1,294
Journal of Youth and Adolescence	2,003
Journal of Adolescence	2,207
Developmental Psychology	5,691
Child Development	7,204
Journal of Adolescent Research	855
**Other**	
Emotion	1,259
Journal of Consulting Psychology	2,614
Social Indicators Research	2,695
Psychological Science	3,496
Journal of Clinical Psychology	5,134
American Psychologist	7,725
Psychological Bulletin	7,963
Personality and Individual Differences	9,216
Journal of Consulting and Clinical Psychology	6,676
Journal of Personality and Social Psychology	9,485

Following the finalization of the journal database, abstracts from each of the 35 journals were downloaded dating back as far as the journal’s inception. The earliest year in the database was 1904 for *Psychological Bulletin*. Abstracts were downloaded for all 35 journals through to end of 2016. If a journal changed its name, the contemporary title was also included to ensure all abstracts were coded as belonging to that journal over time (e.g., *Journal of Research in Character Education* became *Journal of Character Education*; *Professional School Psychology* became *School Psychology*). We encountered a number of duplicated abstracts throughout the data set that were removed (*n*=1,003). Abstracts with no valid year or date of publication, or journal title, were also removed (*n*=14). The final data set consisted of *n*=98,571 abstracts across the 112-year period.

### Step 2: Key Search Terms

After establishing the database, a list of key terms was formed to identify the presence of positive education studies across the 35 journals. A positive education dictionary of terms was built by reviewing eight prior studies that had developed positive education and PP term lists. Prior terms in positive education that were included in the current list included Froh et al.’s (2011) full list of 76 positive terms in school psychology (e.g., flow, mindfulness, savoring, and purpose), a selection of terms from Allen et al.’s (2018a) meta-analysis on school belonging and well-being (e.g., school bonding, teacher, and performance) and a selection of search terms from the SEL meta-analyses by Durlak et al. (2011) and Taylor et al. (2017; e.g., academic achievement, emotions, regulation, and social skills). Beyond the education-specific studies, the *VIA* Institute on Character’s list of 24 character strengths was included (Peterson and Seligman, 2004; e.g., curiosity, love of learning, kindness), as was the list of PP terms generated by Lopez et al. (2006; e.g., empathy, coping, self-efficacy, optimism, and vitality)^5^ and the full list of 233 PP terms developed by Rusk and Waters (2013) in their bibliometric review of PP (e.g., meaning, flourishing, resilience, autonomy, hardiness, and self-awareness). In the case where a term overlapped across the various lists above, the term was included only once in the current dictionary. The draft dictionary was then sent to five experts in the field who reviewed the list and made suggestions for additional terms. The final list of positive education terms totaled 291 and comprised two sublists: one for education and one for PP (see Table 3).

**Table 3:** Words and word stems forming the positive education dictionary.

Education Dictionary		Positive Psychology Dictionary							
academ*	middleschool*	accept	benefi*	concentrat*	energ*	gratitude	judgment	organis*	purpose
adolescen*	numeracy	acceptance	blessing	confidence	engag*	grit	judgment	organiz*	pursuit*
child*	postgrad*	accomplish*	bounce	confident	enhanc*	growth	kind	original*	pwb
class	preschool*	achiev*	brave*	connect	enlighten*	habit*	kindhearted	passion*	qi gong
classmate*	primary	adaptiv*	breathe	connected	ethic*	happier	kindness	pathway*	quality
classroom*	principal	adjusted	broaden*	connection*	eudaemon*	happiness	leader*	patience	rational*
college*	principals	adjustment	buoyan*	conscientious*	eudaimon*	happy	love	patient	reapprais*
curricul*	professor*	admir*	capabilit*	constru*	exceptional*	hardiness	loving*	peace*	recover
educat*	pupil	affect	capital	contemplat*	existential	health	master*	peak	recovery
elementary	pupils	affective	care	control	expecta*	hedonia	meaning*	perfect*	reflect*
faculty	read	affects	caring	cooperat*	extrinsic	hedonic	meditat*	perform*	refocus*
freshman	reader	affirm*	change	cope	fair*	honest*	mental	persever*	refram*
freshmen	reading	agency	character	coping	faith*	honor	mentor*	persist*	regulat*
freshwoman	scholar*	agentic	charit*	counsel*	feeling*	hope*	merciful*	perspective*	relatedness
freshwomen	school*	altruis*	citizen*	courag*	flourish*	humble*	mercy	plan	relational
GPA	secondary	apprais*	civic*	creativ*	flow*	humility	metta	planner	relationship*
grade	senior	appreciate	climate	critical	forgiv*	humor*	mind	planning	resilien*
grades	sophomore	appreciation	coach*	curio*	friend*	identi*	mindful*	plans	resource*
grammar	student*	appreciative	cognitive*	dependab*	fulfil*	imagin*	mindset*	play	respect*
headmaster*	studies	approach	coheren*	determination	fun	inclusi*	mission*	playful*	responsive
instruc*	study*	attachment	coherence	determined	functioning	incremental	modest*	positiv*	salutog*
junior	teach*	attent*	cohesion	diligen*	game*	industr*	mood	posttraumatic	satisfaction
kinder	tertiary	attribut*	cohesive	discipline*	gamifi*	ingenu*	moods	post-traumatic	savor*
kindergarten*	tutee	authenti*	collaborat*	discover*	genero*	insight*	moral*	prayer	sel
kindergartner*	tutor*	autonom*	communal	discretion	genuine*	integrity	motivat*	prosocial*	self*
kindy	undergraduate	aware	communicat*	disengag*	gifted*	intention	noncognitive	pruden*	service*
learn*	universi*	awareness	communities	efficacy	giver	interest*	non-cognitive	psychological	sharing
literacy	upperclass*	beauty	community	effort*	giving	interpersonal*	openminded*	psycho-social	sincer*
literate	varsit*	belief*	compassion*	emotion*	goal*	intrinsic	open-minded*	psycho-social	sisu
math*	young	believe	competenc*	empathy	grateful*	involve*	optimal*	psychotherapy	social*
middle	youth	belong*	competent	empower*	gratif*	joy*	optimis*	ptg	spiritual*

Terms ending with an asterisk are word stems. All words beginning with the prefix before the asterisk are counted as an instance of that word, regardless of the suffix. For example, sincerity and sincere would both be counted as an instance of sincer*.

### Step 3: Filtering the Larger Data Set for Positive Education Abstracts

To be confident that the abstracts included in the final analysis were focused on positive education (as opposed to PP/well-being research not done in education or education research that did not focus on positive topics/well-being), we filtered the data set to include abstracts that had at least one word or word stem from the PP dictionary *and* from the education dictionary (see Table 3). This step took the data set from 98,571 abstracts to 74,496.

#### Analysis Method Part 1: Linguistic Analysis

Linguistic Inquiry and Word Count (LIWC) software was used to generate a proportion of each abstract made up of key terms from the positive education dictionary. LIWC software calculates both a total proportion of the abstract made up of any of the terms in the positive education dictionary, as well as the prevalence for the individual terms comprising the education dictionary, after excluding punctuation (see Pennebaker et al., 2007).

#### Analysis Method Part 2: Human Coding

While the linguistic analysis will examine *what* topics have been studied in positive education over time, we also aimed to examine *how* positive education is being researched. Data were collected on the types of study designs used in positive education (e.g., cross-sectional, longitudinal, and intervention) as well as samples (e.g., students, teachers, and school leaders), research tools (e.g., survey, interview, and classroom observation), the way variables were classified (correlational, independent, and dependent), and settings within which positive education has been conducted. These types of data required detailed human coding. The sample size of 74,496 exceeded the capacity of the researchers to code and, as such, a smaller subset of abstracts was coded. We decided to code the top 2,000 abstracts containing the highest proportion of positive education keywords. In the end, the budget set aside to enable human coding covered slightly more than 2,000 abstracts and the total number of abstracts coded was 2,805. All abstracts were confirmed by coders to have been conducted with student samples and/or in an educational setting (e.g., kindergarten, school, classroom, after-school program, and college/university) and all studied PP topics (note: the abstracts could also contain deficit-based concepts – for example, a study that measures depression together with happiness). At the start of this human coding process, a subsample of 120 abstracts were dual-coded, which involved the second author also coding the same abstracts of a number of all four other coders. This multi-rate data set was the basis for testing inter-rater reliability. Percentage agreement across key nominal research design characteristic variables ranged from 97.7 percent to 100 percent: Cohen’s (1960) kappa ranged from *κ*=0.66–1. Continuous variable agreement was assessed using a two-way mixed, absolute single measures intra-class coefficient (ICC; McGraw and Wong, 1996), and ranged from ICC=0.95 to 0.99, indicating very high agreement.

## Results

### Analysis of Positive Education Term Frequency Over Three Time Frames

The analyses for this review paper focused on three key time frames. First, the data set from 1904 to 2016 was used to track the overall prevalence of aggregated positive education terms in the education and psychology journals for the 112-year period (*n*=74,496). This was done to discover the historical roots of positive education and to examine growth trends in positive education for over a century of research. Researching the overall prevalence of positive education terms across this long time frame provides a big picture of the field.

**Figure 1:**
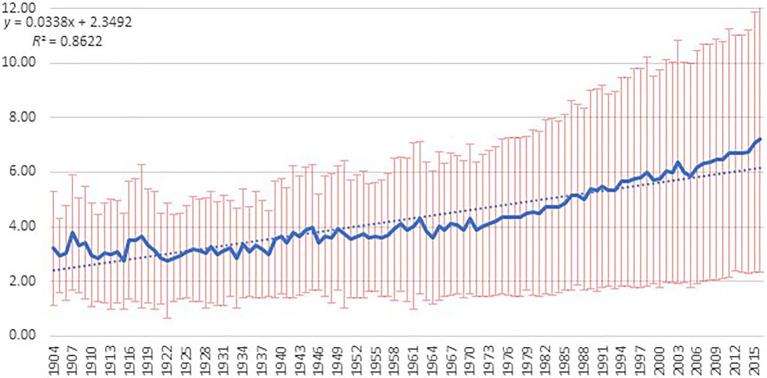
Yearly prevalence of all positive education terms in the complete data set.

Once the long-term aggregated prevalence of positive education terms was identified, the next step was to engage in a more granulated analysis of the specific terms that have gained the most research attention in the field. To make this analysis, the time frame was adjusted to focus on 1950 onwards. The years prior to 1950 were removed owing to the smaller numbers of abstracts from 1904 to 1949 relative to the 1950s onwards, when science in mental health showed a continuous growth. For example, the average number of abstracts per year between 1950 and 1960 was *M*=361.50 (*SD*=37.69), which had increased to *M*=1,902.70 (*SD*=299.70) by the years 2000–2009. Together with the growth in mental health science from the 1950s onwards, the variation in prevalence of key terms from year to year also reduces. Prior to 1950, larger fluctuations in positive education term prevalence across each year in the data are generally evident (refer to the bold, linear trend line in Figure 1), thus making it difficult to establish meaningful and sustained trends prior to 1950. While the smaller numbers and larger variation prior to 1950 allowed for an analysis of aggregated positive education terms, the fine-tuned analysis on specific education terms requires the more consistent data emerging from the 1950s onwards.

When analyzing positive education terms used from 1950 onwards, the data were further was split into two segments: 1950–2008 (*n*=50,938) and 2009–2016 (*n*=17,703). This decision was made due to the official coining of the term and launching of the field of “positive education” by Seligman et al. (2009). By separating the data into these two time frames, insights can be gained as to whether there were any prevalent changes in research topics and directions before and after the official launch of positive education.

Finally, given that the focus of this review paper was on *growth* in positive education, the analysis for all data from 1950 onwards concentrated on the top 20 most prevalent positive education terms as we reasoned that it these that are reflective of growth in the field. This filtering also allowed the large data set to be manageable within the confines and word limits of a peer-reviewed journal article.

#### Time Frame 1: 1904–2016

The first aim of this review paper was to gain a big picture view of positive education research over the past century. The proportion^6^ of positive terms, both in total (i.e., any term in the dictionary; see Table 3) and in specific terms relative to others, were examined across the time frame 1904–2016 (*n*=74,496 abstracts). More specifically, LIWC software generated the proportion of every individual abstract made up of any term in the positive dictionary (the total positive education terms in the abstract) and each specific term, after discounting punctuation terms (Pennebaker et al., 2007). To examine trends over time, mean prevalence for the total and specific terms was calculated for each year in the data set. Then, the mean for each term across all years was generated (i.e., a mean of means). This allowed examination of trends in the psychology and education journals that formed our database across a 112-year period.

As can be seen in Figure 1, the prevalence of positive terms has had a small but steady growth for more than a century. From 1904 to 2016, the linear trend line shows a consistent upward trajectory, explaining 85 percent of variance over time (intercept=2.35; *b*=0.02). The linear trend (dotted horizontal line) shows that by 1952, the prevalence of positive terms consistently sat above the 4 percent mark. From 1982 onwards, positive terms continuously occupied 5 percent of the proportion of research relative to other terms. By 2010, the linear line rose to be consistently above 6 percent. The yearly prevalence line provides further information about how positive terms vary across individual years, and it can be seen that by 2013, the proportion of positive terms in research had climbed above 7 percent. Recall that LIWC produces a proportion and hence controls for the growing corpus of text and suggests more of a growing focus within that larger body of work on positive terms.

#### Time Frame 2: 1950–2008

After gaining a big picture overview of the growth of positive education terms studied in the literature across more than a century of research, we focused the next analyses from 1950 onwards. In order, from most to least prevalent, the top 20 positive education terms studied from 1950 to 2008 were self*; social*; relationship*; perform*; positiv*; emotion*; identi*; control; cognitive*; achiev*; well; health; strateg*; mental; goal*; attent*; motivat*; involve*; change; and affect. Table 4 provides the means and standard deviations for these top 20 positive education terms across this time period.

**Table 4:** Mean and standard prevalence of top 20 terms 1950–2008 and 2009–2016.

1950–2008		2009–2016		Rank order changes for 2009–2016 compared to 1950–2008
Term	Mean and *SD*	Term	Mean and *SD*	
self*	0.29 ± 0.84	self*	0.39 + 1.00	–
social*	0.28 ± 0.78	social*	0.34 + 0.85	–
relationship*	0.18 ± 0.58	relationship*	0.28 + 0.67	–
perform*	0.21 ± 0.59	emotion*	0.26 + 0.84	Increased by three places
positiv*	0.16 ± 0.42	positiv*	0.26 + 0.62	–
emotion*	0.15 ± 0.65	identi*	0.17 + 0.11	–
identi*	0.13 ± 0.50	perform*	0.17 + 0.11	Decreased by five places
control	0.12 ± 0.48	health	0.14 + 0.53	Increased by five places
cognitive*	0.11 ± 0.45	cognitive*	0.13 + 0.45	–
achiev*	0.11 ± 0.50	control	0.13 + 0.49	Decreased by two places
well	0.08 ± 0.26	achiev*	0.12 + 0.50	Decreased by one place
health	0.07 ± 0.45	well-being	0.11 + 0.50	New term
strateg*	0.07 ± 0.42	goal*	0.11 + 0.61	Increased by two places
mental	0.07 ± 0.36	satisfaction	0.11 + 0.52	New term
goal*	0.07 ± 0.38	motivat*	0.10 + 0.46	New term
attent*	0.07 ± 0.42	well	0.09 + 0.26	Decreased by five places
motivat*	0.07 ± 0.42	engag*	0.09 + 0.42	New term
involve*	0.05 ± 0.39	strateg*	0.08 + 0.39	Decreased by five places
change	0.05 ± 0.52	attent*	0.08 + 0.40	Decreased by three places
affect	0.05 ± 0.65	affect	0.08 + 0.40	–

* = word stem.

**Figure 2:**
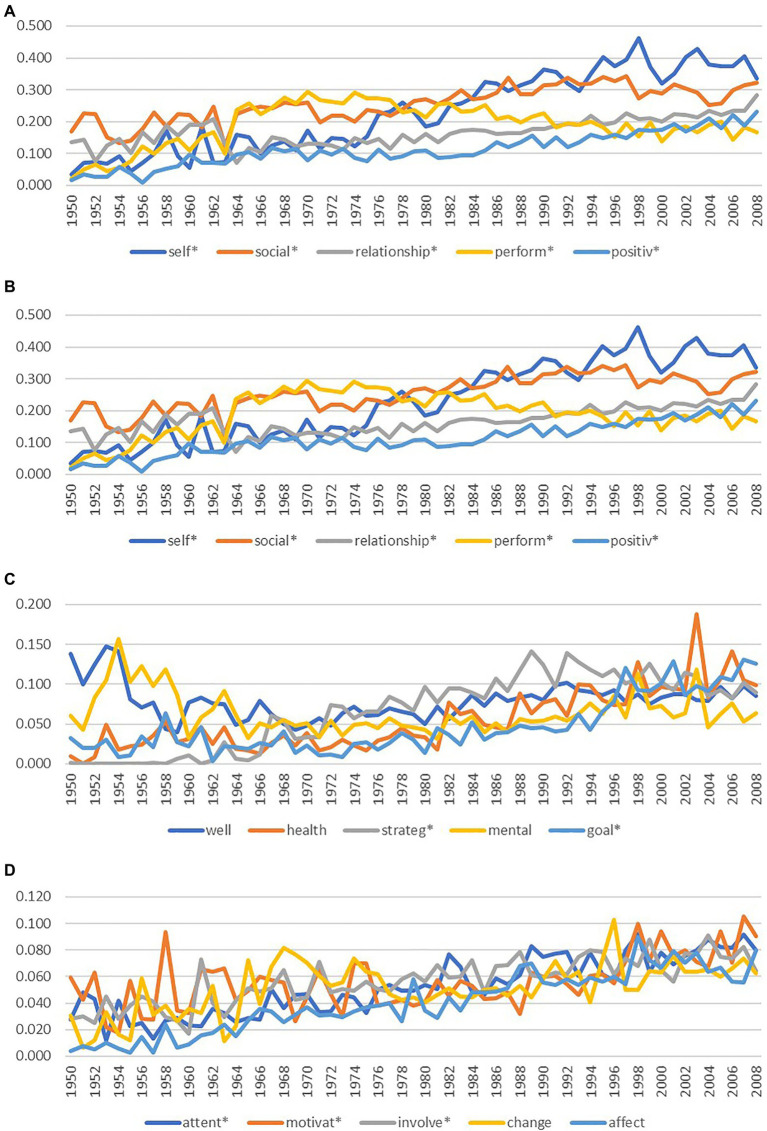
**(A)** Time series of term prevalence for the top five most prevalent positive education terms, 1950–2008. **(B)** Time series of term prevalence for the 6th to 10th most prevalent positive education terms, 1950–2008. **(C)** Time series of term prevalence for the 11th to 15th most prevalent positive education terms, 1950–2008. **(D)** Time series of term prevalence for the 16th to 20th most prevalent positive education terms, 1950–2008.

As well as identifying the mean prevalence, rank order, and relative proportion of the top 20 terms from that time period, we also traced the growth patterns of each of these 20 terms across the 58years. Figures 2A–D present the trends in growth across each term over time. Self* was the only individual term to reach a prevalence above 4 percent. The following four terms started high in the 1950s but there was an observable decline in research from the 1960s onwards: perform*; well; mental; and achiev*. Control was also a term that became less prevalent from the 1990s onwards. All other positive education terms that were in the top 20 from 1950 to 2008 increased over time. Figures 2A–D show that three terms had considerable variability in research prevalence over the 68years: health; motivat*; and change. Research into self and emotion showed a sizeable increase in prevalence in the late 1980s.

#### Time Frame 3: 2009–2016

After analyzing the prevalence and growth of positive education from 1950 to 2008, we focused the next analyses for the period that marked the official launch of positive education by Seligman et al. (2009). The top 20 terms for the period 2009–2016 were self*; social*; relationship*; emotion*; positiv*; identi*; perform*; health, cognitive*; control; achiev*; well-being; goal*; satisfaction; motivat*; well; engag*; strateg*; attent*; and affect. Table 4 provides the means and standard deviations for these top 20 positive education terms. Table 4 also shows the rank order differences in terms as they appeared in the time frame of 2009–2016 compared to the earlier time frame of 1950–2008. Four new terms were present in the top 20 most prevalent terms following the official launch of positive education in 2009: well-being; satisfaction; motivat*; and engag*. The following terms increased in rank order status from 1950–2008 to 2009–2016: emotion*; health; and goal*. It is also of interest to note that for the positive terms that remained in the same rank order between the two time periods, the mean prevalence was consistently higher in 2009–2016 compared to 1950–2008. For example, the mean prevalence rate for self* increased from 0.29 in 1950–2008 to 0.39 in 2009–2016; social* went from 0.28 to 0.34; relationship* from went from 0.18 to 0.28; positiv* went from 0.16 to 0.26, and so on. The following terms decreased in the rank order status across the two time frames: perform*; control; achiev*; well; strateg*; and attent*.

**Figure 3:**
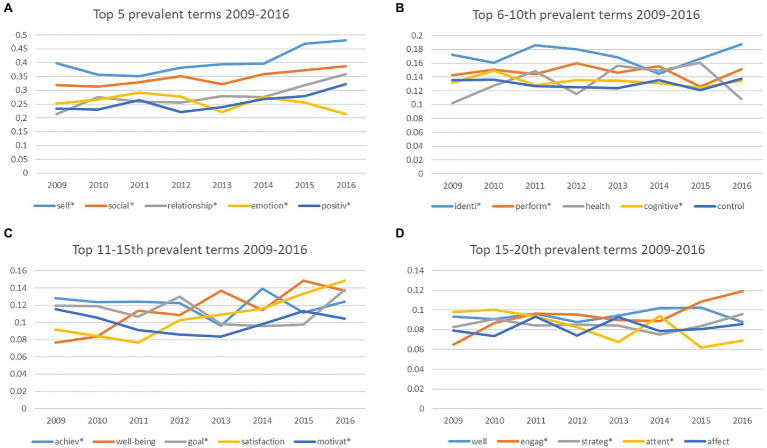
**(A)** Time series of term prevalence for the top five most prevalent positive education terms, 2009-2016. **(B)** Time series of term prevalence for the 6th to 10th most prevalent positive education terms, 2009-2016. **(C)** Time series of term prevalence for the 11th to 15th most prevalent positive education terms, 2009-2016. **(D)** Time series of time prevalence for the 16th to 20th most prevalent positive education terms, 2009-2016.

The growth patterns of each of the top 20 terms in positive education in 2009–2016 can be seen in Figures 3A–D. Self* was the only term that hit a prevalence level of above 4 percent but by 2016, social* came very close, with a prevalence of 3.9 percent and the trend line for social* suggests that it will continue to grow. Ten variables showed slight declines in prevalence in 2009–2016: emotion*; achiev*; well, attent*; mental; motivat*; attrib*; involve*; change; and attribut*. The remaining ten variables showed slight increases in prevalence from 2009 to 2016: self*; social*; relationship*; perform*; positiv*; ident*; goal*; strateg*; health; and affect.

While the focus of this paper is on growth in positive education, it seems worthwhile to also identify under-examined terms in the field. To do this, we combined the two time frames above to create a data set for the period 1950–2016 to identify the terms that had a prevalence of less than 0.001. There were 37 positive education terms that met the baseline cutoff: appreciative; blessing; bounce; brave*; breathe; diligen*; discretion; enlighten*; eudaemon*; gamifi*; giver; hedonia; honor; humble*; ingenu*; kindhearted; merciful*; mercy; metta; non*cognitive; noncognitive; open*minded*; openminded*; patience; planner; psycho*social*; qi.gong; refocus*; savor*; sisu; tai.chi; tai*chi; tenac*; thankful*; valor*; vigor; and zest. The results of this final analysis from the LIWC point to under-investigated topics in the field of positive education and highlight some areas for potential growth in future research.

### Human-Coded Analysis of Research Design Trends in Positive Education Research for the Period 1950–2016

The language analysis above made use of three large data sets and used computer-generated coding to identify the growth and decline of topics studied in positive education over time. By detecting the presence of positive terms since 1904 and discovering the top 20 terms studied in positive education in 1950–2008 and 2009–2016, we can gain a picture about *what* has been studied in positive education. The second phase of the analysis aims to review *how* positive education has been researched over time. To gather accurate data about research designs used in positive education, the analytical method needed to shift from computer-generated analysis to human coding. The abstract data set, which had already been filtered to records that include at least one term from the education and positive dictionaries and from 1950 onwards, was then ordered by the highest prevalence of positive dictionary terms. Five coders then read and identified key research design characteristics in 2,805 abstracts that were confirmed as positive education (2,078 abstracts that focused exclusively on positive topics + 727 studies that involved both positive and deficit-oriented concepts). The research team coded the following: type of paper, research paradigm, design used, tools utilized, samples, research site, research focus, and broad themes.^7^

**Figure 4:**
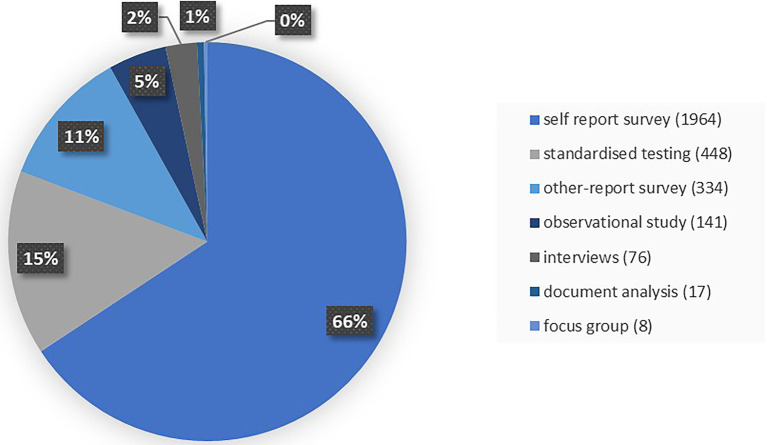
Types of research tools used in positive education, 1950–2016.

With regard to the type of positive education paper, the majority were empirical studies (86.3 percent) followed by theoretical papers (12.4 percent), review papers (0.8 percent), policy analysis (<0.3 percent) and “other” (0.2 percent). Within the empirical studies, the dominant paradigm was quantitative (94.9 percent), with 1.8 percent qualitative and 3.3 percent mixed paradigm (i.e., quantitative and qualitative). There was a variety of research tools used to collect data within the empirical studies, as shown in Figure 4. The three most frequently used tools in positive education from 1950 onwards are self-report, standardized testing, and other-report.

Within the quantitative studies, 88.8 percent were classified as observational, generally correlational studies, where variables were measured and analyzed without researchers attempting to make a change (e.g., an intervention condition). The vast majority of empirical studies were identified as cross-sectional (82.4 percent) with data collected only at a single time point; 26.12 percent were identified as longitudinal designs with data collected more than once; and 8.5 percent were coded as having both a single time point and longitudinal component to the design (some abstracts reported multiple substudies). A wide variety of longitudinal data collection time periods and waves were reported, spanning from a minimum of 1month to multi-year studies spanning the full age period of adolescence. Intervention designs accounted for 11.3 percent of the empirical studies, and within the category of intervention studies, 36.3 percent collected pre-test and post-test data while 40.3 percent included only post-test data.

**Figure 5:**
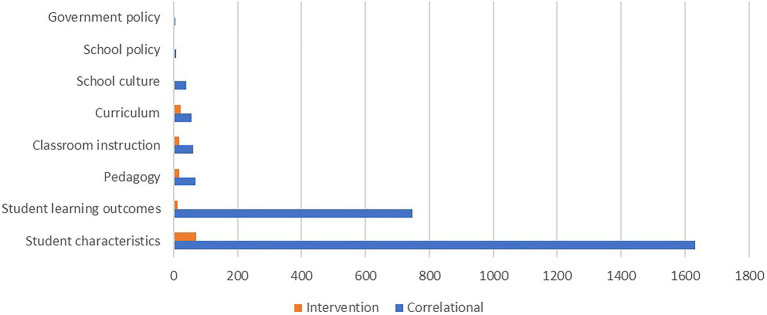
Focus of positive education quantitative research.

Continuing with the analysis of quantitative studies, we sought to explore the broad areas that positive education studies were focusing on. As can be seen in Figure 5, the two most frequent areas of focus in correlational studies were student characteristics (e.g., student motivation, emotional intelligence, self-efficacy, self-esteem, adaptive development, mental illness symptoms, life satisfaction, and coping style) and student learning outcomes (e.g., academic grades, academic self-efficacy, academic goal orientation, engagement, and satisfaction with school). The two most frequent areas of focus for intervention research were student characteristics (e.g., evaluating whether strengths use goes up following an intervention; testing if an intervention can make students more mindful) and curriculum (e.g., assessing the outcomes of a well-being curriculum). School culture, school policy, pedagogy and government policy were underrepresented areas of focus.

Across all papers, students made up the largest sample group (96.5 percent), followed by teachers/school staff (5.0 percent) and parents (3.6 percent) (note: these percentages add up to more than 100% because some studies doubled up on samples, for example a study that included students and parents). Within the studies using student samples, sample sizes ranged from one to 50,000,000 (mode=90; median=1,025; mean=128,035±177,894.8). Age ranged from birth to age 20 (mean=14.19±6.38; only two studies were from birth, one from birth to 54months of age, another from birth to sixth grade), and gender was roughly even (52.96 female; please note: data on nonbinary and trans categories were not provided in the abstracts).

Many abstracts reported a year level as proxy for age. These were coded using the following categories: early learning (ages 0–5), primary (ages 5–12), secondary (ages 13–18), higher education (18+, college/university students), and post-secondary (16+, vocational education and training/apprenticeships/adult education). The results of these categories are presented in Table 5.

**Table 5:** Types of student samples studied from 1950 onwards in positive education.

Educational level	Overall sample (%)	Targeted sample (%)	Convenience sample (%)
Early learning	7.34	5.87	1.47
Primary	23.02	19.04	3.99
Secondary	33.25	25.33	7.92
Higher education	35.76	20.82	14.94
Post-secondary	0.63	0.47	0.16

*Percentage denominator is all studies that reported an educational stage*.

Abstracts were also coded by whether the students formed an integral part of the study (e.g., a targeted student intervention; a study that was specifically looking at the role of positive education in certain age groups; a study examining positive education variables in specific student samples, e.g., students on the spectrum) or whether the students were used merely as a convenience sample. Notably, 843 of the 2,805 abstracts (40 percent) were classified as positive education through the initial filtering process because they studied positive constructs with student samples, but upon human coding, these studies were deemed to be convenience samples because the only educational aspect was the fact that the participants were students. Regardless of whether the sample was targeted or convenient, there is a paucity of research in early learning and post-secondary sector students (adult education, vocational education). As seen in Table 5, the predominance of studies was conducted in higher education (35.76 percent) and secondary schools (33.25 percent) for both the targeted and convenience samples.

Abstracts very rarely identified the school sector or type of school, but where that information was provided, three education sectors were identified: public/government schools were most frequent (0.9 percent), followed by public/independent (0.2 percent) and faith-based schools (0.1 percent). The studies using targeted school students were done in a variety of settings, including the classroom (90 percent), sports field (5 percent), after-school programs (3 percent), and the playground (2 percent).

For the final aspect of the human-coded sample, the two authors of this review paper (who also formed part of the team of five coders) conducted a thematic analysis on all the variables identified in the quantitative empirical studies (3,349 variables total). Due to the very large number of unique variables, a subsample of the most common variables was thematically analyzed to enable exploration of broad areas of focus that have occupied positive education research attention. Thematic analysis of the human-coded data set extends and complements the computer-generated linguistic analysis done on the top 20 positive education terms in the larger data set. Thematic analysis involved grouping similar variables together into broader themes (e.g., “self-esteem” and “domain-specific self-esteem”; or “teacher instruction” and “teacher instruction of behaviors”). Nine broad themes were identified: positive functioning; well-being; ill-being; strengths; connection and belonging; agency; identity and personality; school climate and outcomes; and demographics. Table 6 lists the nine broad themes and provides examples of variables that were placed within each category together with the relative percentage of the themes present in the research since 1950. The three most common themes that have been studied in positive education are school climate and outcomes; identity and personality; and agency. The role of demographics in positive education together with the theme of connection and belonging were the two themes with the lowest percentages.

**Table 6:** Thematic categories studied in quantitative positive education research.

Theme	Exemplar variables	%
Positive functioning	Adaptive development, adjustment to life educational stage, coping style	12.28
Well-being	Positive affect, life satisfaction, happiness, well-being	8.77
Ill-being	Negative affect, aggression, psychopathology symptoms, most commonly anxiety, depression and distress	10.53
Strengths	Persistence, self-regulation, optimism, hope	8.77
Connection and belonging	Parental style, parenting quality, emotional intelligence, empathy	5.26
Agency	Self-efficacy, autonomy, attribution style	14.04
Identity and personality	Self-esteem, Big Five personality factors, attachment style	17.54
School climate and outcomes	Academic achievement, assessment results, cognitive tests, autonomy-supportive teaching, learning goal orientations	17.54
Demographics	Age, gender, stage of education	5.26

The coding rubric asked coders to identify variables as either correlates (no directionality specified), predictor variables (e.g., used as independent variables), or outcome variables (e.g., used as dependent variables), where possible. Interesting trends can be seen in Table 7, including the fact that school climate and outcomes, identity and personality, agency, connection and belonging, and demographic variables were more likely to be used as predictors in positive education research than as correlates or outcome measures. Positive functioning and ill-being were more likely to be used as outcome variables. Strengths were more likely to be studied as correlates, while well-being had an equal percentage use as a correlate and an outcome.

**Table 7:** Thematic categories classified as correlates, predictor variables, or outcome measures.

Theme	Correlates	Predictor	Outcome
	% of top 20 variables		
Positive functioning	5.26	10.53	15.79
Well-being	21.05	0.00	21.05
Ill-being	10.53	0.00	26.32
Strengths	15.79	5.26	5.26
Connection and belonging	0.00	10.53	5.26
Agency	15.79	21.05	15.79
Identity and personality	21.05	26.32	5.26
School climate and learning	5.26	15.79	5.26
Demographic	5.26	10.53	0.00

## Discussion

### Summary of Main Findings

Student mental health has become a priority for many schools across the globe (Seligman and Adler, 2018) and is featured as an explicit goal in educational policy endorsed by key international associations, including WHO, UNICEF, UNESCO, and OECD (WHO et al., 2000; WHO, 2013; OECD, 2015; UNESCO, 2015; WHO and UNESCO, 2021). The COVID-19 crisis has further heightened the importance for schools to find ways to protect and build the mental health of students (WHO, 2020), faculty, and staff given the sharp rise in youth psychopathology (Guo et al., 2020; Marques de Miranda et al., 2020) and teacher stress resulting from the global pandemic (Alves et al., 2020; MacIntyre et al., 2020).

To successfully embed mental health approaches in schools, a robust evidence base is required. Thankfully, this topic has received more than a century of scholarly research. While research into student mental health was initially concerned with the remediation of illness, disorders, and problem behaviors in targeted student samples (Peterson and Park, 2003), the focus has expanded over the decades to also include universal approaches for all students that adopted a prevention orientation in the 1980s and 1990s (Herman et al., 2004) and school-wide initiatives that have adopted a promotion orientation from the 2000s onwards (Waters, 2011).

The advent of PP in the late 1990s has had a valuable input into reshaping remediation approaches, informing prevention approaches, and designing promotion approaches (Shankland and Rosset, 2016; Chodkiewicz and Boyle, 2017). Additionally, PP research has been used to expand the focus beyond students to also include faculty and staff in schools (Kern et al., 2014) and to investigate broader organizational-level approaches that can help schools to create positive systems in classrooms (Allison et al., 2020) and become positive institutions (Waters et al., 2012; Waters and White, 2015). In the current paper, we have included all these approaches within the umbrella term of positive education, and we have argued that education-based mental health research that is positively oriented has existed well before the official launch of the field of positive education in 2009 (Seligman et al., 2009).

This long-standing research history and large evidence base is an asset to schools who are seeking to embed mental health approaches and wish to do so in science-informed ways. An exploration of the large research corpus also provides important information to contemporary positive education researchers about what has been studied and what gaps remain. However, the sheer magnitude of published studies over the past century can make the field difficult to navigate. This is where review papers become valuable because they provide researchers and practitioners with synthesized findings from the field.

Following the official launch of positive education in 2009 (Seligman et al., 2009), 17 review papers have been published that have focused on positively oriented outcomes. These papers have provided important findings and guidance to the field. However, as argued by Chodkiewicz and Boyle (2017, p. 72), “there have been only a limited number of *comprehensive* large-scale reviews to date” (added emphasis). The existing reviews lack comprehensiveness in a number of ways. First, because the vast majority of the existing reviews focused exclusively on intervention studies, information about other types of research designs (e.g., cross-sectional and longitudinal) and inclusion of theory and policy papers is missing. Second, given the small sample sizes drawn upon in the existing positive education reviews – for example, 75 percent contained less than 100 studies; 65 percent contained less than 50 studies; and 41 percent contained less than 20 studies – trends about the larger research patterns have not yet been presented. Third, given that the existing reviews utilized relatively short timelines (the majority of reviews looking at time periods of 10years or less), an understanding of research patterns over longer time frames has not occurred. Finally, the focus of these reviews on single topics and/or a small number of preset topics (constituting 83 percent of the review papers) has resulted in a restricted view of the full scope of positive education research. These gaps have motivated the current “big picture” review study.

We sought to provide a large-scale and long-term “bird’s-eye” view of positive education using significantly bigger samples than prior education reviews (*n* for 1904–2016=74,496; *n* for 1950–2008=50,938; *n* for 2009–2016=17,703) over a time frame that is markedly longer than prior reviews (i.e., 112years). We opted not to focus on preset topics but instead used the data to identify what topics of study have been the most predominant from the 1950s onwards. Furthermore, we cast a broad net to ensure inclusivity of a large range of education-based mental health movements. To balance the breadth of data and associated language analysis of key terms, this review also employed human coding for a more granular analysis of research paradigms, designs, tools, samples, and research sites. By balancing the breadth of language analysis with the depth of human coding, this paper provides robust new insights about *what* has been studied in positive education, and *how* the knowledge has been generated, over a large and historical data set.

The first key finding is that positive education research has existed in some form for more than a century. The start date for analysis in this review paper was 1904, and it can be seen that, even at that time, there were papers being published that contained terms coming from the positive education dictionary. This finding is consistent with Froh et al.’s (2011, p. 119) review of school psychology journals from 1963 to 2008, where they concluded that there has been “a long history of some attention to the study of adaptive and/or optimal development.”

The second key finding arising from the data set is that positive education research has had a slow but steady increase over the course of the 20th century and into the start of the 21st century. The prevalence of positive education terms in peer-reviewed articles rose from 2.9 percent in 1904 to 7.2 percent in 2016. The year 1952 is the point where positive education began to consistently account for 4 percent of research abstract content. This might be a function of the rise of humanistic psychology in the 1950s through to 1970s (Maslow, 1971). Indeed, it was in 1954 that Maslow coined the term “positive psychology” and called for researchers to study positively oriented topics and human potential (Maslow, 1954).

The next notable shift occurred in 1982 when the prevalence of positive education began to steadily account for 5 percent of the published research abstract content. This trend aligns with the rise in the 1980s–1990s of what Kristjánsson (2012, p. 87) refers to as “adaptability psychologies,” such as coping psychology, SEL, and resilience education (Stevens and Pihl, 1983; Creemers and Tillema, 1987).

The third shift occurred in 2010 when positive education began to consistently account for 6 percent of abstract content. This increase occurred the year following the official launch of the field of positive education (Seligman et al., 2009) and was likely influenced by the rise in the 2000s of positively oriented movements in education such as values education (Nielsen, 2005), character education (Berkowitz and Bier, 2005), civics education (Cogan and Morris, 2001), positive youth development (Larson, 2000), and mindful education (Wall, 2005).

It appears that the official launch of positive education in 2009 (Seligman et al., 2009) may have functioned as a catalyst for research growth. For example, while it took 30years for the prevalence of positive education research to jump from 4 to 5 percent (from 1952 to 1982) and 28years for it to jump from 5 to 6 percent (1982–2010), the jump to 7 percent occurred within 5years, in 2015, as shown in the yearly prevalence line of Figure 1. Of course, it could be that the 7 percent prevalence does not remain steady from this point onwards, and future data will be needed to determine whether the upward trend continues, accelerates toward an exponential trend, stabilizes, or declines. However, the fact that the yearly prevalence line consistently sits above 6.5 percent from 2010 onward, rises to above 7 percent for 2015 and 2016 and does not show any backsliding from 2009 onwards, points to the idea that the launch of positive education as a formal field has functioned as a catalyst for research growth. Further support for this idea can be seen in the review findings of Rusk and Waters (2013), who conducted a large bibliometric analysis of the broader field of PP (launched in 1999) and found in the subsample of education journals that positive education papers had tripled.

Differences in topics studied prior to and after the official launch of positive education also point to the launch as a catalyst. Comparison of the top 20 most widespread terms from the period 1950–2008 to the period 2009–2016 shows that after the official launch of the field, four new positively oriented topics gained prominence: well-being; satisfaction; motivat*; and engag*. Additionally, three positively oriented terms that were being studied prior to the launch rose in prevalence and rank order after 2009: emotion*; health; and goal*. Moreover, for the positive terms that were equally ranked across both time periods, the mean prevalence was consistently higher in 2009–2016 compared to 1950–2008 (e.g., see the mean increases for positive*, relationship*, and social*) showing that more research was being conducted on these positively oriented topics and that they accounted for a higher proportion of the overall literature.

Such trends point to the idea that the official launch of positive education as a field may have helped to mobilize the growth of positively oriented education research and to place attention on some of the newer PP constructs. Oades et al. (2011a), Vella-Brodrick (2011), and Shankland and Rosset (2016) have also made this claim. It is important to note that the rise of new positive topics since the launch of positive education is not at the expense of some of the time-honored mental health and education research topics, such as affect, self, health, and achievement, all of which have remained predominant from the 1950s through to 2016. The ongoing research emphasis on some of these longer-term constructs led Kristjánsson (2012, p. 86) to ponder whether positive education is merely “old wine in new bottles” but it is probably more apt to think about this as an enlargement of the wine cellar that is stocked with older, more established labels and has added a new range of wines.

It is important to make clear that we do not seek to nullify earlier education movements that have been positively oriented by suggesting that the official launch of positive education has been a catalyst. We do not suggest that it has been the sole catalyst – merely that is has been a supporting factor (just as humanistic and adaptive psychologies seem to have prompted further lines of topical literature). Indeed, as stated above, the data reveal that mental health and positively oriented research in education have been around for a very long time in many different forms. We have reasoned that this research can retrospectively fall under the umbrella of positive education. In this way, we have suggested that positive education had a history before Seligman et al. (2009) labeled it. It is this history and long research momentum that set the stage for the official launch of the field by Seligman and his colleagues. Based on the rising mean prevalence of positive topics within education-oriented abstracts, this launch poised researchers to place a greater focus within scientific publications on positive education topics and widen the scope of study to new positively oriented topics. In other words, by providing a label and a mission, the official launch of positive education seems to have accelerated what already existed and triggered a greater focus and breadth of research in education-based mental health.

Stepping back to look at the big picture, one can see that the percentage of positive education research present across the 35 education, psychology, and well-being journals reviewed in the current paper is still reasonably small (7 percent), as is the mean prevalence of the top 20 terms in the field, despite the steady increases in proportion. There is still room for considerable growth in positive education and the broader impact it can have across the fields of education, psychology, and well-being.

One finding of interest was the sizeable number of constructs that have been the focus of study in the broader PP literature over the past two decades that have not yet received attention within education. Indeed, there were 37 terms from the positive education dictionary that had a mean prevalence of less than 0.001, including several of the *VIA* character strengths such as bravery, zest, and forgiveness (mercy). This was also the case for many terms pertaining to mindfulness (e.g., breathe, refocus, openminded, and savor), and several pro-social terms (e.g., giver, kindhearted, and psycho*social). Froh et al. (2011, p. 119) found the same results in their school psychology review and concluded that there has been “inattention to many relatively new PP constructs that have been shown to be of importance to the well-being of adults and children.”

Within the positive education areas that have been studied, the language analysis revealed what terms were most often studied. To complement this, the human coding phase of this review sorted the study variables into nine broad research themes that have been present in positive education research since 1950. Again, these results show a blend between the more established “stalwart” topics in the field, such as agency, identity, and personality with the addition of newer foci such as well-being and positive functioning. As with the language analysis that showed that pro-social constructs had low prevalence in positive education, the theme of connection and belonging was equal lowest in the human-coded results (together with demographics) suggesting that this is a fruitful area for growth in the field. Strengths is another area that came out to be reasonably low in the human-coded themes, with some strengths also featured in the terms that had a prevalence of less than 0.001 percent of the data set.

In addition to identifying *what* has been studied in the field of positive education over time, the current review paper also shed light on *how* positive education has been studied by analyzing the types of paper, research paradigms, designs, tools, samples, and research sites from 1950 onwards (human-coded sample; *n*=2,805 abstracts). Analyzing the research trends in how positive education research has been conducted, where it has been conducted and who it has been conducted with highlights gaps in the field. It also provides important information that can be used to understand the common findings as well as put caveats in place on the overarching claims being made about positive education. For example, the fact that comparatively little research has been conducted in the early learning years means that claims about the effectiveness of positive education cannot yet be extended to younger children. Additionally, the fact that the impact of demographic factors were the equal lowest area of focus in positive education, and considering that we did not find any abstracts allowing for nonbinary gender categories, suggest that that caution is required when assuming that the general positive education findings will apply to minority groups (e.g., racial minority, gender and sexual minority, low socio-economic status, regional versus metro, and so on).

Of the types of papers published in positive education, empirical studies were by far the most common (79.5 percent) and this is consistent with the initial calls from founders in PP for the field to distinguish itself from other earlier positively oriented movements by focusing on empirical science (Seligman and Csikszentmihalyi, 2000; Peterson, 2006). Yet, the bird’s eye view provided by the current analysis could be used to suggest that the pendulum has swung too far and that there is a need for more theoretical papers to help the field grow and expand. Moreover, given that review papers and policy analysis accounted for only 1 percent of the publications, there is room for growth with these two types of contributions. Of course, these results could be a reflection of the 35 journals that formed the database, and a different journal selection may have resulted in a higher proportion of theoretical, review, and policy papers being found. However, given that the vast majority of the 35 journals had been utilized by former review papers (albeit with smaller subsamples), and the well-being/PP journals were also endorsed by the five experts consulted, it is likely that these journals well represent the types of positive education papers being published. As such, the trend of few theoretical, review, and policy papers is most likely valid and, while it is somewhat expected, expansion of these methodologies may help enrichen the field.

It is interesting to note that the majority of positive education review papers published since 2009 have focused on intervention studies (76 percent; see Table 1) and yet intervention studies only accounted for 11.3 percent of the papers published in the field. Interventions were most often conducted in the classroom (90 percent) but were also implemented in after-school programs, the playground and the sports field – the latter three indicating that positive education has a role to play in shaping the broader contexts and culture of schools beyond the classroom.

That said, context did not feature heavily as a research focus in the positive education data set. As shown in Figure 5, within the quantitative research, student characteristics, an individually oriented topic, were the area of focus that received the highest percentage of research in both the correlational (62.5 percent) and intervention research (49.65 percent). Student learning outcomes, another individually oriented area, received the highest percentage of research in correlational research (28.6 percent). The more contextually oriented areas of study, such as policy, culture, and classrooms, had a much lower percentage in the quantitative positive education research. These results point to a criticism that positive education is too often decontextualized (Ciarrochi et al., 2016) and supports calls to extend beyond intra-individual factors to contextual, cultural, and system factors that shape positive outcomes for individuals, groups, and institutions (Waters et al., 2015b; Roffey, 2017; Owens and Waters, 2020). Allen et al. (2016) argue that schools operate as nested systems that incorporate many levels of influence. Ecological models suggest that context affects well-being at distal and proximal levels (Bronfenbrenner, 1977). Distal aspects of context can include school climate and school policy – both of which are pointedly underrepresented in the positive education literature in the current data set. Proximal contextual factors include the classroom environment (teacher pedagogy and instruction as well as the curricula taught in class) and significant relationships (friendships, parent-child relationships), which, again, did not feature strongly in the data set. Contextual ecological models of well-being in schools have recently received research attention (see Waters et al., 2019, 2021a; Allison et al., 2020; Kern et al., 2020), but these papers were published after 2016 and, thus, were not part of the data set used in the current paper. While the criticism of decontextualization is beginning to be addressed, more attention is needed to context in positive education.

Stepping beyond research at the individual level to investigate context and systems requires “epistemological broadening, both in terms of scope and methodologies” (Lomas et al., 2020, p. 2) particularly the use of qualitative and/or mixed-methods research approaches – both of which were scant in the positive education data set. The neglect of qualitative paradigms in positive education aligns with a criticism directed toward the broader field of PP (Hefferon et al., 2017; Lomas et al., 2020). Qualitative research opens up inductive approaches that play an important role in theory building. For example, Brunzell et al.’s (2016, 2018) qualitative study on the way teachers find meaning when working with traumatized students led to the Trauma-informed Positive Education (TIPE) model. Moreover, by asking different questions of established educational phenomena, qualitative research opens a portal to new and extended understandings of existing positive education constructs. For example, Howells’ (2014) qualitative research on gratitude in schools as an “action” expanded the existing quantitative approaches on gratitude in schools as an emotion, thereby opening the door to a wider range of action-based gratitude interventions. The field would benefit from a higher proportion of qualitative research and by using a more diverse set of research tools, such as case studies, interviews, focus groups, and document analysis (which currently only account for 7 percent of the research tools used). Other diverse methods such as Delphi studies (Elmendorf and Song, 2015), implicit approaches (Williams et al., 2017), and the use of student drawings (Waters et al., 2021b) are making their way into positive education, indicating that the field is slowly extending its boundaries beyond quantitative methods that focus on individual-level research to qualitative approaches that embrace context and greater complexity.

### Study Limitations

The findings discussed above must be considered within the limitations of the current review study. To address the gaps that exist in other positive education review papers, we designed a study that used large data sets, with no predetermined topics, over very long time periods. The strength of large data sets is that of generalization, but this also presents a challenge in how to find ways to meaningfully synthesize copious volumes of information. To trace whether positive education had grown over time and identify the most prevalent topics, computer-based language analysis was applied. This allowed a big picture view to be provided on an analysis of terms. However, the gains in breadth from language analysis are counterbalanced by the lack of detail. An abstract may have contained a term from the positive education dictionary – for example, hope – but the prevalence analysis could not tell us whether that term was the focus of the study or was more of an “incidental” or nonessential word used in the abstract. For example, it could be that the authors ended the abstract with a sentence stating that they *hoped* the research inspired future studies. To try to address this limitation, for the human coding component we filtered the data from 1950 onwards and sorted by abstracts that contained the highest proportion of positive education terms (i.e., the abstracts contained multiple positive education terms), thus reducing the risk of including abstracts that contained one-off, spurious positive education terms.

The decision to focus on the abstracts with higher numbers of positive terms was also made because the focus of the current paper was on growth in positive education. To identify common trends in how positive education research has been conducted since 1950, it was important to ensure that the data set represented the abstracts that had the highest prevalence of positive education terms rather than the abstracts that had fewer. If this project had more funding, it would have been interesting to also code the abstracts that had the lowest proportion of positive education terms to see whether there were differences in research paradigms, designs, topics, tools, settings, and so on – this set of abstracts may be examining different topics, using more varied research designs, and derive from a higher proportion of nonempirical papers.

We aimed to offset the limitations of language analysis by also conducting human coding on a relatively large sample of 2,805 abstracts. The inter-rater reliability statistics across the team of five coders was high. Human coding allowed for more detail to be identified about how positive education has been conducted since 1950. These results provided valuable information but also identified some findings that conflicted with those identified in the language analysis. For example, word count analysis showed that the terms social* and relationship* were among the highest terms present in the data set from 1950 onwards. Moreover, the term social* was trending to reach 4 percent in the 2009–2016 data set. Yet, when variables identified were categorized into broader themes, the theme “connection and belonging” was the least prevalent. Term prevalence analysis results seemed to differ then from the human-coded identification of variables most studied. This shows how different subsamples within the larger data set provide unique results given that the term analysis was completed on the large LIWC data set, while the theme analysis was performed on a smaller human-coded subsample of abstracts. This also shows how the level of detail used can influence the results found and reminds the reader to consider the limitations of this review (and of other review papers) when drawing conclusions about the field.

One criticism that has been directed at educational, development, and psychology research is the over-reliance on convenience samples (Nielsen et al., 2017; Zhao, 2020). This is also the case in positive education, where 40 percent of studies using student samples coded by the research team were identified as convenience student samples rather than purposeful samples. This could certainly be seen as a limitation of the current review paper and a limitation that is reflective of the broader body of positive education and, indeed, psychological science. It also begs the question of what constitutes positive education. Is positive education any positively oriented/mental health research that includes student samples or must it use a purposeful student sample that occurs within educational contexts?

Finally, as already outlined, the data set of journals used may be considered a limitation. The choice to use selected journals rather than a science database (e.g., PsychLIT, or ERIC, Web of Science) was made because the topic – positive education – straddles the intersection between two broad fields and, thus, the use of selected journals allowed for a more targeted database. We followed the protocol of other researchers who had conducted reviews on positive education by using selected journals to form the database (Durlak et al., 2011; Froh et al., 2011; Kristjánsson, 2012; Taylor et al., 2017), and we double-checked the list of 35 journals with five experts in the field. However, if we had selected different journals, the results may have changed. This brings us back to the point of making methodological decisions that allowed for insights to come from a very large data set while not making the data so large that is it unmanageable. Along similar lines, the analysis focused on abstracts rather than the full papers to allow for manageability. Using the full paper may have generated higher term prevalences for Figure 1 and would have shown a higher proportion of terms. Thus, by using only the abstracts, the growth statistics calculated in this review are likely to be a conservative estimate. It could also be that using the full papers may have generated a different list of the top 20 terms. However, we reasoned that it is the abstracts that provide the most focused text of the paper and, thus, provide the most accessible and succinct view of what the study is researching.

These findings also add to calls for researchers to report studies comprehensively and succinctly in abstracts. A recent review benchmarked the completeness of randomized controlled trials (RCTs) in oncology abstracts against Consolidated Standards of Reporting Trials (CONSORT) reporting requirements (Sivendran et al., 2015) and found they included a median of nine of 17 key details, with certain features with excellent coverage (eligibility criteria, interventions, endpoints), and others more poorly represented (trial design description, blinding, registration). We were unable to find an equivalent to Sivendran et al.’s (2015) benchmarking review paper that has performed an analysis of abstract completeness in the fields of PP or education, but it would be fair to say that there were abstracts in the current data set that were not fully comprehensive, and this may have shaped the findings we obtained. As such, we call for researchers in positive education to be as comprehensive as possible in their future publications. As noted by Sivendran et al. (2015), this is critical given that abstracts are often publicly available where full manuscripts are not and, as such, are likely to inform evidence-based practice.

## Conclusion

Positive education has great potential to improve the lives of students and others involved in education systems, in its aim to foster capacities needed to maintain mental health promote and well-being across the lifespan. The long-term presence of research in this field together with its more recent growth, both in size and breadth, has contributed much knowledge. The current review shows that positive education research is indeed growing. However, our findings also suggest that this growth is limited in some respects by a lack of diversity in certain paradigms, research designs, samples, methods, and settings. This large-scale review identified certain “blind spots” in positive education research, such as an over-reliance on observational, cross-sectional, self-report survey designs with high school and university students (often using convenience samples) and a relative absence of research in the early years and primary-aged students. Future positive education researchers are advised to consider the unique potential contribution to knowledge by using studies that are intervention-based with longitudinal designs and that are undertaken with purposeful samples. Moreover, qualitative research and studies that investigate context and systems in positive education will help to expand the field.

## Data Availability Statement

The raw data supporting the conclusions of this article will be made available by the authors, without undue reservation.

## Author Contributions

LW and DL worked together on this paper in a 50/50 contribution, co-designed the methodology, and worked on multiple drafts of the full and final paper. LW conceived of the idea for a large scale bibliometric review of the field of positive education and led the introduction and discussion section. DL led the data management, statistical analysis and results section of the paper. All authors contributed to the article and approved the submitted version.

## Conflict of Interest

The authors declare that the research was conducted in the absence of any commercial or financial relationships that could be construed as a potential conflict of interest.

## Publisher’s Note

All claims expressed in this article are solely those of the authors and do not necessarily represent those of their affiliated organizations, or those of the publisher, the editors and the reviewers. Any product that may be evaluated in this article, or claim that may be made by its manufacturer, is not guaranteed or endorsed by the publisher.

## Footnotes

^2^As shown in Table 1, the use of targeted journals to find relevant papers was used by 29 percent of former positive education review papers.

^3^Journals from the broader fields of positive psychology and well-being science were not included by Froh et al. (2011), who used four school psychology journals; Kristjánsson (2012) reviewed one journal – the *Education Psychologist*; Durlak et al. (2010, 2011) as well as Taylor et al. (2017) focused more specifically on SEL as opposed to the broader field of positive education and while their databases also included some prevention-oriented journals there was no inclusion of promotion-oriented positive psychology and well-being journals. We rectified this in our list by adding positive psychology and well-being journals to the database.

^4^Two experts were known by their publications in top-ranked journals: two were editors of positive psychology or education journals and one was the chair of the Positive Education Division of the International Positive Psychology Association.

^5^The Lopez et al. (2006) list was also included in Froh et al. (2011).

^6^The *proportion*, rather than the *raw number*, of positive terms was calculated to control for the trend of growing research output (i.e., the increasing numbers of abstracts in the data set for each passing year).

^7^As outlined in the “Materials and Methods” section, the abstracts selected for part 2 of the data analysis were those identified with the highest prevalence of positive education terms. To identify common trends in how positive education research has been conducted since 1950, it was important to ensure the data set represented the abstracts that had the highest prevalence of positive education terms rather than the abstracts that had fewer positive education terms. If the research had more funding, it would have been interesting to also code the lowest 2,000 abstracts to see whether there were differences in research paradigms, designs and so on compared to the abstracts high on positive education.
